# Gold nanoparticle assemblies stabilized by bis(phthalocyaninato)lanthanide(III) complexes through van der Waals interactions

**DOI:** 10.1038/srep03758

**Published:** 2014-01-20

**Authors:** Yuki Noda, Shin-ichiro Noro, Tomoyuki Akutagawa, Takayoshi Nakamura

**Affiliations:** 1 Graduate School of Environmental Science, Hokkaido University, N10W5, Kita-ku, Sapporo 060–0810, Japan; 2 National Institute of Advanced Industrial Science and Technology (AIST), AIST Tsukuba Central 4 and 5, Tsukuba 305–8562, Japan; 3 Research Institute for Electronic Science, Hokkaido University, N20W10, Kita-ku, Sapporo 001–0020, Japan; 4 Institute of Multidisciplinary Research for Advanced Materials, Tohoku University, 1–1 Katahira, 2-Chome, Sendai 980-8577, Japan

## Abstract

Gold nanoparticle assemblies possess diverse application potential, ranging from industrial nanotechnology to medical biotechnology. Because the structures and properties of assemblies are directly affected by the stabilization mechanism between the organic molecules serving as protecting ligands and the gold nanoparticle surface, it is crucial to find and investigate new stabilization mechanisms. Here, we report that π-conjugated phthalocyanine rings can serve as stabilizing ligands for gold nanoparticles. Bis(phthalocyaninato)lutetium(III) (LuPc_2_) or bis(phthalocyaninato)terbium(III) (TbPc_2_), even though complex, do not have specific binding units and stabilize gold nanoparticles through van der Waals interaction between parallel adsorbed phthalocyanine ligands and the gold nanoparticle surface. AC magnetic measurements and the electron-transport properties of the assemblies give direct evidence that the phthalocyanines are isolated from each other. Each nanoparticle shows weak electronic coupling despite the short internanoparticle distance (~1 nm), suggesting Efros–Shklovskii-type variable-range hopping and collective single-electron tunnelling behaviours.

Gold nanoparticle (AuNP) assemblies have attracted considerable attention because of their outstanding properties, which are applicable to devices in fields such as electronics^1^, magnetics^2^ and plasmonics^3^. Various methodologies have been developed to produce assembled structures, such as polymer-mediated structures^4^,^5^, structures with different particle size^6^ and lithographically-defined template structures^7^,^8^. One of the most sophisticated methods to construct a AuNP assembly structure is to use DNA as a building block, which enables arrangement of nanoparticles in three dimensions^9^. However, the physical properties can also be controlled by modifying the stabilization mechanism, as well as the electronic and/or magnetic properties of the organic molecules protecting nanoparticles. The physical properties of gold nanoparticle assemblies, as well as the stability and size distribution of the particles, are determined by stabilization mechanisms through interaction with organic molecules. Interactions between gold nanoparticles and molecules are mainly classified into two types: covalent bonding and physisorbed interaction between the particle surface and molecules. Covalent bonding protection has been mainly used to synthesize gold nanoparticles because the chemical bonding between the gold atom and the molecule, such as Au–S, provides enough stability to allow the facile synthesis of thermally stable particles with a narrow size dispersion. On the other hand, physically protected nanoparticles have attracted practical interest from the view-point of weak interactions between molecules and nanoparticles. For example, catalytic reactions^10^ and cell recognition^11^ take advantage of surface accessibility of reactive species or cells. Fabrication of an electrical circuit through a solution-based process can also be achieved using weakly stabilized metal nanoparticles because the removability of the surface ligand permits a decrease in the sintering temperature, which is a crucial factor from the view-point of energy cost^12^, and field-effect transistor performance^13^. Physically stabilized nanoparticle assemblies are also favourable for achieving single-electron tunnelling^14^ because physisorbed molecules prevent strong electronic coupling between nanoparticles and/or nanoparticle–molecule coupling that cause an increase in conductance and a decrease in the tunnelling decay constant^15^.

Several approaches have been attempted to stabilize gold nanoparticles through physisorption using specific binding groups such as carboxylates, amines and pyridine. For example, Griffin and Fitzmaurice found that 4-(dimethylamino)pyridine was adsorbed normal to the surface of the gold nanoparticle through electrostatic interaction with the nitrogen atom of the pyridine ring^16^. An aqueous polymer, poly(*N*-vinyl-2-pyrrolidone), was also able to stabilize gold nanoparticles through multiple co-ordination of the nitrogen and oxygen atoms of the pyrrolidone units^4^. Unexpectedly, thienyl^8^ or acetylthio group^17^ substitution of porphyrin derivatives at meso positions stabilized gold nanoparticles through electrostatic or multiple Au-S interactions which is much weaker than thiol-gold covalent bond. However, we could not completely eliminate the possibility of covalent bond formation between gold surface with these molecules at the chalcogen-atom sites.^18^ Motivated by these results, we adopted π-conjugated macrocycles as stabilizers of gold nanoparticles. Stabilization of gold nanoparticles with a π-conjugated macrocycle reduces the particle–molecule or particle–particle distance and permits incorporation of molecules with high dielectric constants between gold particles while keeping electronic coupling between them weak. Therefore, we can expect, for example, to observe collective single-electron tunnelling^8^ or nanoscale inductor or capacitor behaviours^3^.

Here we present the novel gold nanoparticle assemblies which stabilized by neutral bis(phthalocyaninato)lanthanide(III) (LnPc_2_) complexes through v. d. Waals interaction (Figure 1). These complexes have two distinct features: 1) the complexes have no specific binding units able to connect onto the gold surface covalently. In addition, electrostatic interaction between neutral molecules and nanoparticles should be small. Instead, the conjugated π system of phthalocyanine ligands is expected to act as the binding site. 2) Electronic and magnetic properties of the complexes can be controlled easily. For example, the energy level of the frontier orbitals can be modified by introducing electron-withdrawing/donating groups to the phthalocyanine ligand or by extending the π system. Moreover, the magnetic properties of the complexes drastically change by changing the lanthanide ion from diamagnetic and paramagnetic to that of a single-ion magnet. Here, we employ two neutral complexes: bis(phthalocyaninato)lutetium(III) (LuPc_2_) and bis(phthalocyaninato)terbium(III) (TbPc_2_). The former is diamagnetic (Lu^3+^:^1^S_0_), whereas the latter is known to be a single-ion magnet, whose magnetic moment arises from Tb^3+^ (^7^F_6_). By employing LnPc_2_ complexes as a protecting ligand for gold nanoparticles, inter-nanoparticle distance was sufficiently close while the electronic nanoparticle-nanoparticle and nanoparticle-complex interactions remained weak. Such weak electronic interactions within the assembly prevented the disappearance of plasmonic or semiconducting properties^19^. This implies that the LnPc_2_–AuNP assemblies provide the opportunity to control the physical properties by molecular design rather than by the arrangement of nanoparticle arrays. These gold nanoparticle assemblies will open up new applications such as the realization of the biomimesis of an artificial neural network brain whose elements are integrated through weak interactions.

## Results

### Characterization of gold nanoparticle assemblies stabilized by bis(phthalocyaninato)lanthanide(III) complexes

Figure 2 shows TEM images of LuPc_2_–AuNP and TbPc_2_–AuNP. Both LuPc_2_–AuNP and TbPc_2_–AuNP were observed as black aggregates with a size of several hundred nm. Each aggregate was composed of monodispersed Au particles with diameters of about 4 nm connected with each other (Figure 2 inset). Isolated particles were not observed. Due to very poor solubility of LnPc_2_–AuNPs in ordinary solvents, we could not obtain, NMR, electronic (absorption, diffuse reflectance) and vibration spectra (IR, Raman).

In the aggregates, nanoparticles were arranged randomly, showing no periodic arrangements. Random particle arrangements have often been observed in aggregates connected by strong interactions between particles, such as nanoparticles covalently linked by alkane dithiol^20^. On the other hand, regularly arranged nanoparticles with closed packing structures have been achieved through weak van der Waals interactions working between particle to particle surfaces driven by, for example, the hydrocarbon chain of dodecanethiol^21^, in which the nanoparticles can rearrange to energetically minimum arrangements. The random arrangements of both LuPc_2_–AuNP and TbPc_2_–AuNP would be caused by π−π interaction of phthalocyanine ligands adsorbed on the surface of the gold nanoparticles.

We estimated the number and the molecular orientation of LnPc_2_ molecules adsorbed on the gold nanoparticles. By avoiding the aggregated area observed in the TEM images, we estimated the diameter of the gold nanoparticles as 3.7±1.2 nm and 4.2±1.4 nm for LuPc_2_-AuNP and TbPc_2_-AuNP, respectively (see Supplementary Fig. S1). Based on the assumption that each gold nanoparticle is composed of dense-packed Au atoms^22^ with 7 shells, we estimated that the number of atoms in one gold nanoparticle was 1.4×10^3^ gold atoms, which can be obtained from the formula Σ10n^2^+2, with n being the atomic shell number. It leads to a theoretical diameter of 3.8 nm, which is close to the experimentally estimated diameter. A 3.8 nm diameter spherical gold nanoparticle has a surface area of ca 45 nm^2^. Elemental analysis of LuPc_2_–AuNP and TbPc_2_–AuNP showed C:H:N = 7.44%:0.97%:1.44% and 7.11%:0.55%:1.95%, respectively, indicating that the ratios of the complexes and gold atoms are 1:46 and 1:49 for LuPc_2_ and TbPc_2_, respectively. Therefore, each gold nanoparticle should have ca 29–30 molecules of LnPc_2_ on the surface. The number of adsorbed LnPc_2_ molecules was nearly the same irrespective of the lanthanide ions. In the case of TbPc_2_–AuNP, a static magnetization measurement provided information on the number of TbPc_2_ molecules adsorbed on a gold nanoparticle. The *χ_m_T* value around room temperature was obtained as 12 emu K mol^−1^ by assuming 30 molecules on each 3.8 nm gold nanoparticle (see Supplementary Fig. S2) which value is in good agreement with that of the free trivalent Tb ion (11.81 emu K mol^−1^)^23^. These results give further evidence that the estimated number of molecules adsorbed on one gold nanoparticle and the diameters of nanoparticle are appropriate.

**Figure 1:**
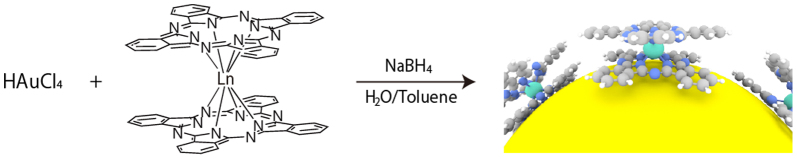
Synthesis scheme of bis(phthalocyaninato)lanthanide(III) protected gold nanoparticles (LnPc_2_–AuNP, Ln = Lu, Tb).

The cross-sectional area of a LnPc_2_ molecule for the flat-on configurations was estimated to be 1.63 nm^2^ from a molecular model^24^ constructed using Mercury (Ver. 2.3) and ImageJ (Ver. 1.43u). The surface area of ca 45 nm^2^ for the 3.8 nm gold nanoparticle can accommodate 28 molecules of LnPc_2_ in flat-on configurations, which agrees well with the result of elemental analysis. A schematic representation of LnPc_2_–AuNP is shown in Figure 1. The poor solubility of the assemblies should be explained by the strong π–π interaction between phthalocyanine ligands paralley absorbed on gold nanoparticles surface.

X-ray photoelectron spectroscopy (XPS) analysis for LuPc_2_–AuNP and TbPc_2_–AuNP assemblies also demonstrated (1) the existence of LnPc_2_ complexes and gold nanoparticles in assemblies and (2) neutral electronic states of the complexes on the gold nanoparticle surface (see Supplementary Discussion S1, Figs S3 and S4 and Tables S1–S3).

### AC magnetic properties of TbPc_2_–AuNP

To characterize the magnetic properties of TbPc_2_ as a single-molecule magnet in the gold nanoparticle assemblies, alternating current (AC) magnetic susceptibility and magnetization of TbPc_2_–AuNP were studied. Figure 3a shows the temperature-dependent AC susceptibility of TbPc_2_–AuNP assemblies with various AC-field frequencies. As the temperature decreased, *χ_M_*′*T* decreased abruptly, where *χ_M_*′ is the real part of the AC susceptibility, clearly indicating the phase lag of the magnetization response from the external magnetic field. Each peak position of the *χ_M_*″/*χ_M_* vs. *T* plot, where *χ_M_*″ and *χ_M_* are the imaginary part of the AC susceptibility and DC susceptibility, respectively, provides the precise temperature where the *χ_M_*′*T* decrease is observed. Ishikawa et al.^25^ reported that TbPc_2_ follows the Orbach process, which gives a barrier height (*Δ*) in a specific temperature range, where *Δ* is the energy to invert the magnetic moment of Tb^3+^. Because *Δ* depends on the strength of the ligand field, we can estimate the oxidation state of the Pc ring from *Δ*. The value of *Δ* was estimated from the slope of the Arrhenius plot of ln*τ^−^*^1^ vs. 1/*T*,
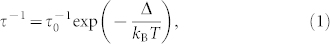
where *τ* is the magnetization relaxation time calculated from the angular frequency *ω* of the applied AC field^25^ and *k*_B_ is the Boltzmann constant. In Figure 3b, linear behaviour was observed above 40 K, indicating that the Orbach process was dominant. The estimated barrier height *Δ* was 472 cm^−1^, which is close to that of the neutral state (*Δ* = 260^25^, 410^26^, 550^27^ cm^−1^ for monoanionic, neutral and monocationic TbPc_2_, respectively). The blocking temperature (*T*_B_) corresponding to an anisotropy barrier was about 47 K at 1042 Hz, which is also close to the neutral state (*T*_B_ = 40^25^, 50^26^, 52^27^ K at 997 Hz for monoanionic, neutral and monocationic TbPc_2_, respectively). Deviation from the Orbach process at temperatures lower than 40 K is an intrinsic TbPc_2_ property^25^, and did not originate from interaction between TbPc_2_ molecules and the gold nanoparticle.

Figure 3c shows the magnetization curves measured at 1.8 K for bulk [TbPc_2_]^0^ and TbPc_2_–AuNP. No hysteresis behaviour was found for bulk [TbPc_2_]^0^, as reported previously^25^. A clear butterfly-type hysteresis behaviour was observed for TbPc_2_–AuNP. Such a butterfly-type hysteresis behaviour was observed when TbPc_2_ was diluted at a concentration of 2% in a diamagnetic matrix (YPc_2_)^25^ or attached to single-walled carbon nanotubes through π–π interactions^28^. Intermolecular interactions between TbPc_2_ molecules were reduced in TbPc_2_–AuNP, as with the diluted [TbPc_2_]^0^ in a YPc_2_ matrix.

The Cole-Cole plot for bulk TbPc_2_ (Fig. 3d), measured at 2 K, shows a single semicircle shape. On the other hand, the Cole-Cole plot for TbPc_2_-AuNP shows two relaxation phases corresponding to the high and low-frequency regions of applied AC frequency. These results strongly suggest that the environment of a TbPc_2_ molecule in TbPc_2_-AuNP is different from the environment in bulk TbPc_2_. Based on of the opened hysteresis loops (Fig. 3c right) and the Cole-Cole plots, we concluded that the TbPc_2_ molecules absorbed on the gold nanoparticles in TbPc_2_-AuNP were relatively isolated from each other.

### Electron-transport properties of LuPc_2_–AuNP and TbPc_2_–AuNP assemblies

The weak interaction between a gold nanoparticle and the complexes strongly affected the electron-transport properties. The resistance of LuPc_2_–AuNP at room temperature (*R*_R.T._) was smaller than that of TbPc_2_–AuNP by one order of magnitude (Table 1), which originates from the difference in the sample volume. The sample volume will determine the number of electron paths described in terms of percolation^29^ which affects the absolute resistance value, but will not affect the conduction mechanism. Figure 4 shows the temperature-dependent normalized resistance (*R/R*_R.T._) of the assembled structures of LuPc_2_–AuNP and TbPc_2_–AuNP. The *R/R*_R.T._ of both assembly structures from room temperature to approximately 220 K obeyed an Arrhenius-type temperature dependence (see Supplementary Fig. S5). The activation energies of the nanoparticle assemblies were almost the same, irrespective of the adsorbed molecules; 39.8 meV and 41.8 meV for LuPc_2_–AuNP and TbPc_2_–AuNP, respectively. The activation energy is strongly influenced by the particle size and distance between the nanoparticles^30^. The similar activation energies for the LnPc_2_–AuNPs are in good agreement with the TEM measurements and elemental analysis, where the monodispersed nanoparticles are separated by LnPc_2_ in a flat-on conformation in both LuPc_2_– and TbPc_2_–AuNP.

On lowering the temperature below 220 K, the *R/R*_R.T._ deviated from an Arrhenius plot. The values of *R/R*_R.T._ became proportional to exp(*T^−^*^1/2^) in the temperature range from 82–15 K and 123–20 K for TbPc_2_–AuNP and LuPc_2_–AuNP, respectively (see Table 1 and Supplementary Fig. S6). Below 20 K, *R/R*_R.T._ of LuPc_2_–AuNP became temperature independent down to 1.8 K, while that of TbPc_2_–AuNP continued to obey *R/R*_R.T._ ∝ exp(*T*^−1/2^).

The semiconducting charge transport behaviours in nanoparticle assemblies reported so far often obeyed the variable-range hopping (VRH) model,
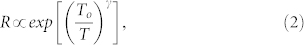
where *T*_0_ is a constant and the value of *γ* ranges from 0 to 1. *γ* = 1 represents Arrhenius behaviour. If strong Coulomb interactions exist, then the system obeys a *γ* = 1/2 law, which is independent of the dimensionality, and is known as ES-VRH^31^. Therefore, we consider that *R/R*_R.T._ in the temperature range below 220 K should obey ES-VRH-type behaviour.
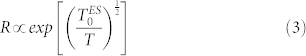
Here, *T*_o_^ES^ represents
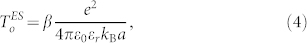
where *k*_B_ is the Boltzmann constant, *e* is the electron charge, *ε*_0_ is the dielectric constant in a vacuum, *ε_r_* is the relative dielectric constant of the surrounding medium (*ε_r_* = 30 and 28 for LuPc_2_ and TbPc_2_, respectively, obtained from dielectric measurements) and *a* is the localization length of the electronic wave functions. We evaluated the electronic coupling strength between gold nanoparticles using the localization length *a* estimated from the specific value of *T*_o_^ES^. The coefficient β depends on the dimensionality of the conducting electrons (for two and three dimensions, β = 6.5 and 2.8, respectively)^32^. We used 6.5 for β because we found that the two-dimensional pathway was used from analysis of the *I–V* characteristics, as mentioned below. Table 1 summarizes the parameters of the ES-VRH models. From equations (3) and (4), we obtained *a* = 1.18 nm and 0.71 nm for LuPc_2_–AuNP and TbPc_2_–AuNP, respectively, which were smaller than the diameter of the gold nanoparticles. A consideration of weakly protected gold nanoparticle feature based on the localization length *a* will be described in discussion section.

The values of the hopping distance (*r_hop_*)^30^,^33^ of the conduction electrons can be estimated from
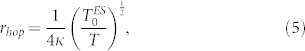
where *κ* is the tunnelling decay constant, which is the reciprocal of the localization length *a* (*κ* = *a*^−1^). The *r_hop_* values are summarized in Table 1. Those for both LuPc_2_–AuNP and TbPc_2_–AuNP were equal at 1.5 nm, well matched with the nearest-neighbour particle distance, at the temperatures where the transport mechanism crossed over from an Arrhenius-type thermally activated hopping to an ES-VRH-type hopping mechanism around 123 K and 82 K for LuPc_2_–AuNP and TbPc_2_–AuNP, respectively. The results indicate that the cross-over from Arrhenius- to ES-VRH-type conduction was decided by the *r_hop_* distance of the transport electrons.

**Figure 2:**
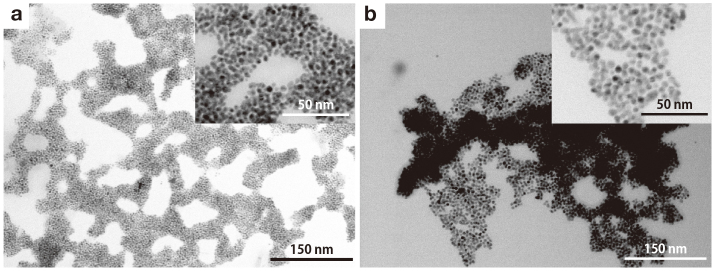
TEM images of (a) LuPc_2_–AuNP, (b) TbPc_2_–AuNP. Enlarged images are in the upper right corner.

**Figure 3:**
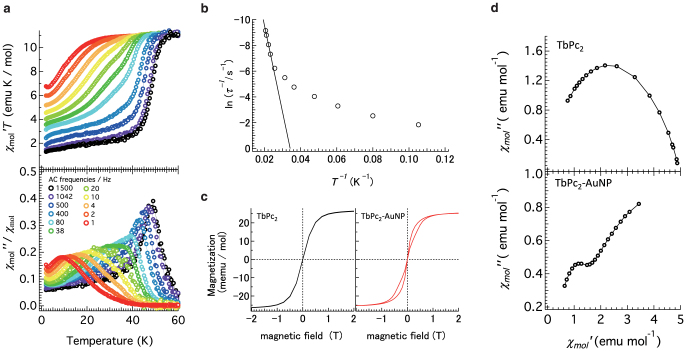
AC magnetic properties of TbPc_2_–AuNP. (a) Plots of temperature and AC frequency dependence of *χ*_mol_′*T* and *χ*_mol_″/*χ*_mol_, where *χ*_mol_′, *χ*_mol_″ and *χ*_mol_ are in-phase AC, out-of-phase AC and DC molar magnetic susceptibilities, respectively. The measurements were performed in a 7 Oe AC oscillating magnetic field. (b) Temperature dependence of the magnetization relaxation rate. The solid line denotes linear fits given by equation (1) in the temperature range above 40 K. (c) Magnetic hysteresis circles of bulk TbPc_2_ and TbPc_2_–AuNP measured at 1.8 K with an average sweep rate of 10 Oe/s. (d) Cole-Cole plot at 2 K for TbPc_2_ and TbPc_2_-AuNP, respectively.

The *I*–*V* characteristics of the LuPc_2_–AuNP below 20 K (Fig. 5) were explained by the model of Middleton and Wingreen (the MW model)^34^, which is based on electron transfer through the nanoparticle assembly taking into account randomly charged nanoparticles separated by tunnel junctions. The *I*–*V* characteristics of the model are described by
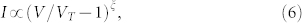
where *V_T_* is the Coulomb-blockade threshold voltage and *ξ* is the dimensionality of the current pathway, which depends on the structure. The values of *ξ* = 1 and 5/3 were calculated for infinite one-dimensional and two-dimensional arrays, respectively, and numerical studies indicated that 2.2<*ξ*<3.0 for multilayered arrays^35^,^36^. The model reproduced the *I*–*V* characteristics of LuPc_2_–AuNP at 10 K well (Fig. 5a), indicating that electrons tunnel through the nanoparticles with the Coulomb blockade. The value of *ξ* remained approximately 2.5 irrespective of the temperature from 1.8 K to 10 K (Fig. 5b). This indicates that the dimensionality of the tunnelling electron flow is not strictly two-dimensional because the LuPc_2_–AuNP structure is composed of the random arrangements of nanoparticles demonstrated by TEM measurements (Fig. 2). The reasonable value of *ξ* implies that the contribution from the ES-VRH-type hopping mechanism was completely suppressed. The threshold voltage below 10 K was in the range 0.4–0.6 V and decreased linearly with increasing temperature at 20 K keeping the same *ξ* value. This implies that the tunnelling electrons were under competition from the thermal fluctuation and the Coulomb effect below 20 K. The reason that the threshold voltage changed and *ξ* remained unchanged with increasing temperature above 10 K is because the thermal fluctuation disturbed the Coulomb effect while the dimensionality of the electron flow was maintained^37^.

## Discussion

We consider van der Waals interactions to be the predominant interaction between LnPc_2_ and the surface of the gold nanoparticles for the following reason: XPS analysis showed the molecular structure of both TbPc_2_ and LuPc_2_ phthalocyanine ligands to be unchanged before and after stabilization of the gold nanoparticle. Additionally, the neutral electronic state was confirmed by the blocking temperature (T_B_) value, which was estimated by the AC magnetic properties. Komeda et al.^38^ concluded through STS measurements that neutral TbPc_2_, with an unpaired electron in the π-orbitals of the phthalocyanine ligand, interacted weakly with a flat Au (111) substrate surface. Based on the XPS and AC measurement results, v. d. Waals interactions are the dominant interactions between the lanthanide complex and the gold nanoparticles.

Weakly protected gold nanoparticle by LnPc_2_ through v. d. Waals interactions showed a small localization length *a* and the *I-V* characteristics reproduced by a MW model for electron transport measurements. The localization length *a* demonstrates the weak interaction between a gold nanoparticle and the complexes. In nanoparticle arrays, the magnitude of *a* should be equivalent to the size of each nanoparticle^39^,^40^, and the larger values of *a* imply a stronger coupling between nanoparticles. For example, Romero et al.^41^ reported the localization length *a* of 3.5 nm, which is comparable to the diameter for oleic acid-ligated PbSe nanoparticles (2.75 nm), which increased in size to approximately 102 nm after vacuum annealing, where the diameter then remained constant. They suggested that the origin of the large *a* was due to strong coupling between PbSe nanoparticles through reduction of the barrier heights by annealing. In LuPc_2_–AuNP and TbPc_2_–AuNP, each nanoparticle packed densely in the assembled structure and the internanoparticle distance is short (~1 nm), as seen from the TEM images (Fig. 2). Despite that, the localization lengths *a* for LuPc_2_–AuNP (1.18 nm) and TbPc_2_–AuNP (0.71 nm) were significantly smaller than the particle diameter (about 4 nm). This strongly suggests that the gold nanoparticles are in a weak electronic coupling state with each other because both of the LnPc_2_ molecules adsorbed on its surface through weak v. d. Waals interactions.

The *I*–*V* characteristics of the LuPc_2_–AuNP showed the non-liner behaviour with threshold voltages described by the MW model. If the electronic coupling between the nanoparticles became strong, such as systems with covalent bonds between the gold nanoparticles and a saturated^40^ or conjugated molecule^42^, the conduction behaviour tends to obey a coherent tunnelling model. Coherent tunnelling provides a conduction channel at low applied bias through several junctions resulting in the power law *I–V* behaviour with no threshold voltage^40^. The *I*–*V* characteristics obeyed the MW model closely, indicating the weak electronic coupling in the LuPc_2_–AuNP assembly.

Such a weak v. d. Waals interaction prevents the strong hybridization between the molecular orbitals of the complex and the Fermi level of gold^38^. We confirmed the small localization length and threshold voltage behavior by electron transport measurements. These results demonstrate that the transport electrons are confined within the nanoparticles because the complexes adsorbed on the nanoparticles act as an insulating barrier to prevent the electronic coupling between adjacent nanoparticles. This means that the electronic structures of the “gold nanoparticle-complex-gold nanoparticle” system were isolated from each other. Taking these results into account, the interaction between the LuPc_2_–AuNP and the gold surface is considered weak.

**Figure 4:**
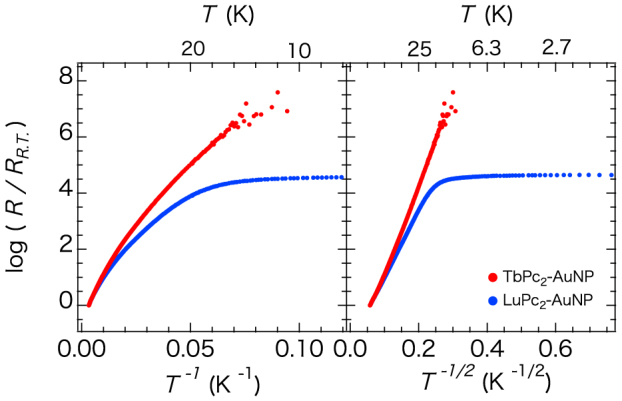
Normalized resistance of LuPc_2_–AuNP and TbPc_2_–AuNP assembly structures plotted vs. *T*^−1^ and *T*^−1/2^.

**Figure 5:**
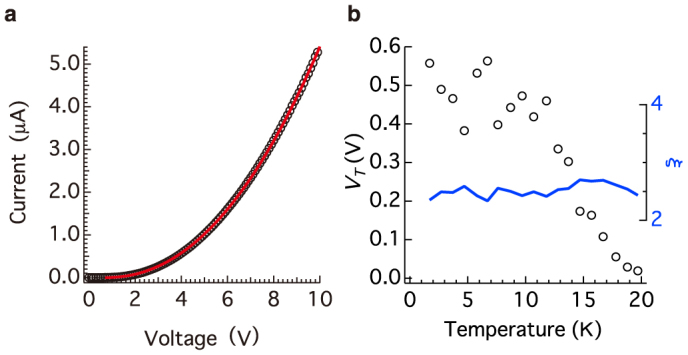
(a) *I*–*V* characteristics of a LuPc_2_–AuNP assembly at 10 K. The red line denotes the MW model given by equation (3). The parameters *V_T_* and *ξ* were 0.48 V and 2.4, respectively. (b) Temperature dependence of the parameters *V_T_* and *ξ*.

In contrast, the TbPc_2_–AuNP assembly did not show a temperature-independent behaviour of *R/R*_R.T._ down to the lowest temperature (Fig. 4). Additionally, the *I*–*V* characteristics did not obeyed the MW model (see Supplementary Fig. S7). As for the electron transport through TbPc_2_, the Kondo effect, which originates from the correlation between the π-spin of the phthalocyaninato (Pc) ligand and the tunnelling electron, has been reported by Komeda et al.^38^. Vincent et al.^43^ utilized the Kondo effect derived from the Pc ligand to detect the quantum tunnelling of the magnetization (QTM) of a single Tb^3+^ ion. Both examples suggest that the direct interaction between the transport electron and the Tb^3+^ electronic state did not occur. The electronic state in both LuPc_2_–AuNP and TbPc_2_–AuNP is neutral. Therefore, they have a π-spin on the phthalocyaninato ligand. Because similar activation energies were observed above 20 K for both LuPc_2_–AuNP and TbPc_2_–AuNP, the difference between the conduction behaviors at lower temperature could not be attributed to the difference in the number of conduction paths between electrodes or the difference in sample dimensions. One of the possible causes of this result is a direct correlation between the tunnelling electron and the spin states of the Tb^3+^ ion, although this requires needs further investigation.

In conclusion, we synthesized and characterized gold nanoparticle assemblies protected by bis(phthalocyaninato)lanthanide(III) complexes, which were stabilized through v. d. Waals interaction between the π-conjugated macrocycle of the phthalocyanine ligand and the gold nanoparticle surface. The assembly structures were composed of monodispersed gold nanoparticles with a diameter of about 4 nm. The phthalocyanines were adsorbed with a parallel conformation on the gold surface and both the LnPc_2_ and the gold nanoparticles were in the neutral states. TbPc_2_ showed the magnetic properties of single-molecule magnets with a clear hysteresis loop in TbPc_2_–AuNP, showing that the molecules were isolated from each other on the gold surface. Even though LnPc_2_ did not have a specific binding unit capable of covalent bonding or electrostatic interaction, it stabilized the gold nanoparticles through v. d. Waals interaction between the π-conjugated macrocycle of the phthalocyanine ligand and the gold nanoparticle surface. The weak interaction between the gold nanoparticle and the phthalocyanines affected the electron-transport properties. The electron-transport mechanism for both assembled structures changed depending on the temperature. By decreasing the temperature from R.T. to 1.8 K, they showed an Arrhenius-type thermally activated hopping, ES-VRH-type hopping and collective single-electron tunnelling. The geometrical equivalences between the LuPc_2_–AuNP and TbPc_2_–AuNP assembled structures were demonstrated by the activation energy and hopping distance obtained from the Arrhenius model and ES-VRH model, respectively. The localization length *a* of the transport electron in the ES-VRH model was significantly smaller than the diameter of the gold nanoparticles, indicating weak electronic coupling between the gold nanoparticles in LnPc_2_–AuNP. Collective single-electron tunnelling was observed in the LuPc_2_–AuNP assembled structure, indicating that the electrons were confined within each gold nanoparticle because of weak electronic coupling between gold nanoparticles at lower temperatures. Such a Coulomb-blockade feature will collaborate with the emerging field of bioelectronics, for example, the biogating behaviour of coupling with living cells^44^ and/or the stochastic resonance to construct artificial neuron communication^45^.

## Methods

### Synthesis of LuPc_2_–AuNP and TbPc_2_–AuNP assemblies

The neutral complexes of LuPc_2_ and TbPc_2_ were synthesized according to the literature^46^. Gold nanoparticles protected by LuPc_2_ or TbPc_2_ were synthesized using a two-phase system, as described by Brust et al.^47^. A 10 ml aqueous solution of HAuCl_4_·3H_2_O (35 mg, 0.09 mmol) was mixed with 30 ml toluene solution of tetraoctylammonium bromide (TOAB). The two phases were stirred until all of the HAuCl_4_ had transferred into the toluene phase. A 30 ml toluene solution of LuPc_2_ or TbPc_2_ (104 mg, 0.09 mmol) was added, and the mixture was stirred for 15 min at 10°C. Then, 1 ml aqueous solution of NaBH_4_ (33 mg, 0.3 mmol) was slowly added. After stirring for a further 10 min, the toluene phase was separated and concentrated. Ethanol was added, and the resulting precipitate was washed with ethanol, toluene and chloroform to obtain gold nanoparticles protected by LuPc_2_ or TbPc_2_ (referred to as LuPc_2_–AuNP and TbPc_2_–AuNP, respectively) (Fig. 1).

### Materials characterizations

TEM measurements were carried out using a Hitachi HD-2000 TEM employing an acceleration voltage of 200 kV. Samples for the TEM measurements were prepared on a carbon-coated Cu grid (Okenshoji Co., Ltd., Japan) by drop-casting of a toluene solution of the LuPc_2_–AuNP and TbPc_2_–AuNP suspension, and then the toluene was allowed to evaporate. The XPS measurements were carried out using a JPS-9200 electron spectrometer (JEOL, Japan) equipped with a rotating anode Al Kα X-ray source. The pressure of residual gases in the analyser chamber during XP spectra acquisition was 4×10^−6^ Pa. The peak positions were calibrated using the binding energy of Au (4f_7/2_ = 84.0 eV) and In (3d_5/2_ = 433.9 eV). The spectra were fitted by symmetric Voigt functions after subtracting the Shirley-type background. The temperature-dependent AC and DC magnetic susceptibility, hysteresis loop measurements and Cole-Cole plot were carried out using a Quantum Design MPMS-XL5 magnetometer for a TbPc_2_–AuNP powder sample. For electron-transport measurements, toluene suspended solutions of TbPc_2_–AuNP or LuPc_2_–AuNP were drop-cast onto gold electrodes (thickness of 50 nm and a gap of 30 μm) formed on SiO_2_ substrates. After the solvent was evaporated in a vacuum at room temperature, temperature-dependent DC resistance and current–voltage (*I*–*V*) characteristics were measured using a picoammeter voltage source (Keithley Model 6487) with a constant bias voltage in the range of −5 to +5 V in a PPMS (Quantum Design Japan) over the range 300–1.8 K. Measurements were carried out on at least five samples to confirm the reproducibility.

## Author Contributions

Y.N. conceptualized the research and conducted all of the experiments. T.A. was responsible for construction of the electrical transport measurement system. Y.N., S.N. and T.N. discussed the experiment. Y.N. and T.N. did the main part of the preparation of the manuscript.

## Supplementary Material

Supplementary Information Supplementary Information

## Figures and Tables

**Table 1:** Room temperature resistance and fit parameters for TbPc_2_–AuNP and LuPc_2_–AuNP obtained from *T*^−1^ and *T*^−1/2^ fits using Arrhenius and ES-VRH models, respectively

	*R*_R.T._/Ω	Arrhenius-type/K	*E_A_*/meV	ES-VRH-type/K	*T*_0_^ES^/K	*ε*_r_	*a*/nm	*r*_hop_/nm
TbPc_2_–AuNP	33356	R.T. – ca 220	41.8	123–20	5450	28	0.71	1.5 (82 K) – 3.4 (15 K)
LuPc_2_–AuNP	4393	R.T. – ca 220	38.2	82–15	3075	30	1.18	1.5 (123 K) – 3.3 (25 K)
