# Supplementary material for: Gold nanoparticle assemblies stabilized by bis(phthalocyaninato)lanthanide(III) complexes through van der Waals interactions
Source: Sci Rep. 2014 Jan 20;4:3758. doi: 10.1038/srep03758 (PMC3895872; doi:10.1038/srep03758)
Supplement: Supplementary Information [file srep03758-s1.pdf]

Supplementary Information

Gold nanoparticle assemblies stabilized by bis(phthalocyaninato)lanthanide(III) complexes through van der Waals interactions

Yuki Noda[1,2*], Shin-ichiro Noro[1,3], Tomoyuki Akutagawa[4] and Takayoshi Nakamura[1,3*]

[1]Graduate School of Environmental Science, Hokkaido University, N10W5, Kita-ku, Sapporo 060-0810, Japan
[2]National Institute of Advanced Industrial Science and Technology (AIST), AIST Tsukuba Central 4 and 5, Tsukuba 305-8562, Japan
[3]Research Institute for Electronic Science, Hokkaido University, N20W10, Kita-ku, Sapporo 001-0020, Japan
[4]Institute of Multidisciplinary Research for Advanced Materials, Tohoku University, 1-1 Katahira, 2-Chome, Sendai 980-8577, Japan

Supplementary Figures S1–S7

Supplementary Tables S1–S3

Supplementary Discussion S1

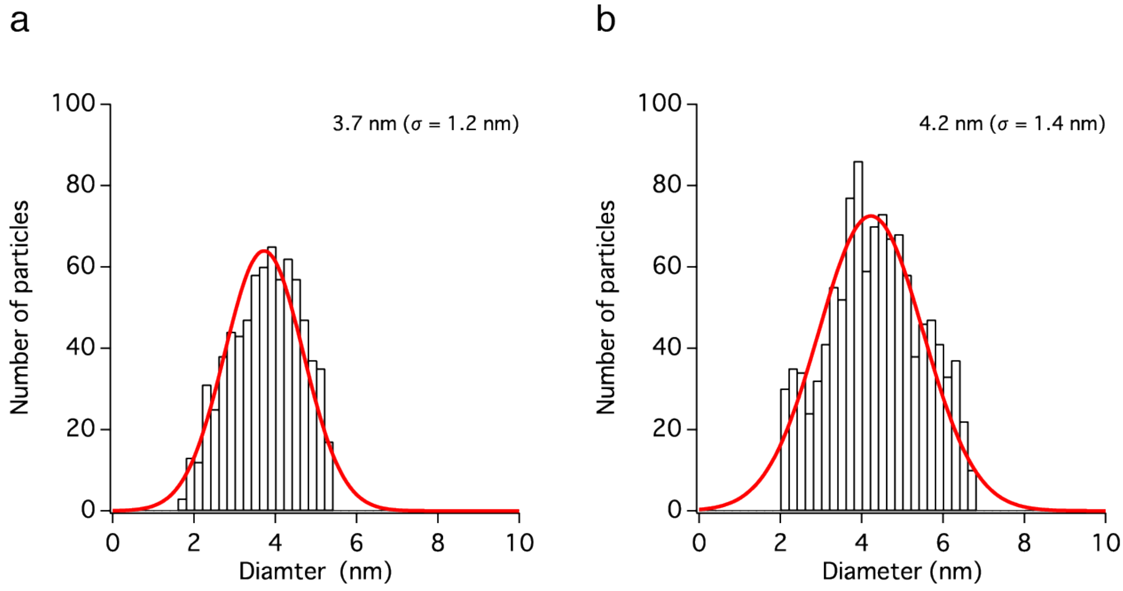

Figure S1| Size distribution of (a) $LuPc_2$–AuNP and (b) $TbPc_2$–AuNP asseblies.

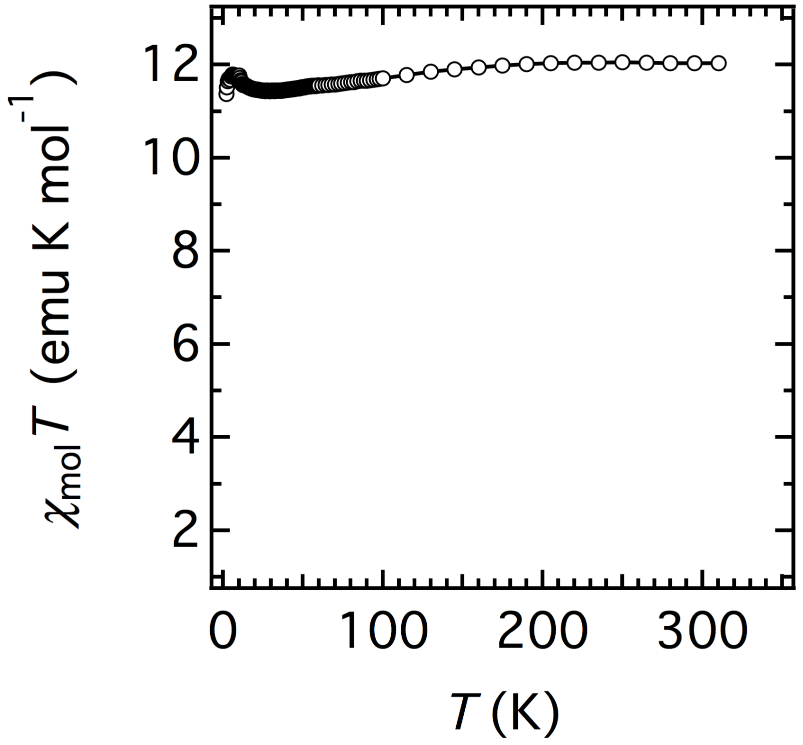

Figure S2| Temperature dependence of $\chi_{mol}T$ for $TbPc_2$–AuNP.

Discussion S1 | XPS analysis for $LuPc_2$–AuNP and $TbPc_2$–AuNP assemblies.

XPS measurements were used to determine the electronic states of the complexes and gold nanoparticles. Electronic spectra (absorption, diffuse reflectance) and vibration spectra (IR, Raman) did not give adequate signals because of the strong aggregation of particles and the small number of molecules. Here, we will discuss only $LuPc_2$–AuNP because the $TbPc_2$–AuNP signals showed essentially the same features as those of $LuPc_2$–AuNP. The N 1s emission peaks in the spectrum of bulk $LuPc_2$ were consistent with those previously reported[1] in terms of binding energy (BE) and intensity (see Fig. S2a), which was deconvolved into one main peak and one shake-up satellite peak (A and A′ in Fig. S2a, respectively). For $LuPc_2$–AuNP, the BE of the main peak of the N 1s emission (A in Fig. S2b) coincided with that of bulk $LuPc_2$, indicating the existence of $LuPc_2$ in $LuPc_2$–AuNP, while the BE of the satellite peak (A′ in Fig. S2b) shifted about 1 eV to higher energy than that of bulk $LuPc_2$. In addition, the satellite/main peak intensity ratio increased by about a factor of two, from 0.6 for bulk $LuPc_2$ to 1.0 for $LuPc_2$–AuNP (Table S1). The C 1s emission peaks showed the same tendency as that of N 1s. For $LuPc_2$–AuNP, the chemical shift between the two main peaks A (porphyrinic carbon) and B (benzenic carbon) was about 1.1 eV, which agrees with bulk $LuPc_2$ and a previous report[2] indicating that the aromatic π system of $LuPc_2$ was maintained in the gold nanoparticle assembly structure. Satellite peaks of the bulk $LuPc_2$ C 1s emission (A′ and B′ in Fig. S2c) were observed at 1.9–2.1 eV higher than the main peaks (A and B). This energy corresponds to that of the Q-band of the Pc rings (668 nm, 1.86 eV)[2,3], indicating that the observed satellite peaks can be accounted for as shake-up-type satellites. For $LuPc_2$–AuNP, the peak shift between the main and satellite peaks increased to 2.3–2.6 eV. In addition, the satellite/main peak area ratio increased by a factor of two, from 0.3 to 0.6 (Table S1). The Au $4f_{7/2}$ and $4f_{5/2}$ emissions of $LuPc_2$–AuNP shown in Figure 2e appeared at 83.9 eV and 87.6 eV, and are assigned to Au(0).

The shake-up satellite peaks (A′ in Fig. S2a, A′ and B′ in Fig. S2c) observed in bulk $LuPc_2$ can be interpreted as intrinsic satellites that are derived from the π–π* transition of Pc's Q-band and have been reported for other molecules, such as TPP[2], Pc[4] and PcFe derivatives[5]. On the other hand, satellite peaks observed in $LuPc_2$–AuNP showed two different features: 1) higher BE shift and 2) changes in the satellite/main peak area ratio. The origin of the satellite peaks of $LuPc_2$–AuNP should be energy loss of photoelectrons[6]. The energy-loss-type satellite is derived from inelastic scattering of photoelectrons travelling from inside to the surface of the sample. In that case, the satellite peaks appear in a higher energy region than the main peaks with broad full width at half maximum (FWHM). Moreover, the change in satellite/main peak area ratio is the same independent of the element. These features agree well with the satellite peaks that appeared in $LuPc_2$–AuNP. The energy loss of the photoelectron should be caused by inelastic interaction between the photoelectron and gold nanoparticles.

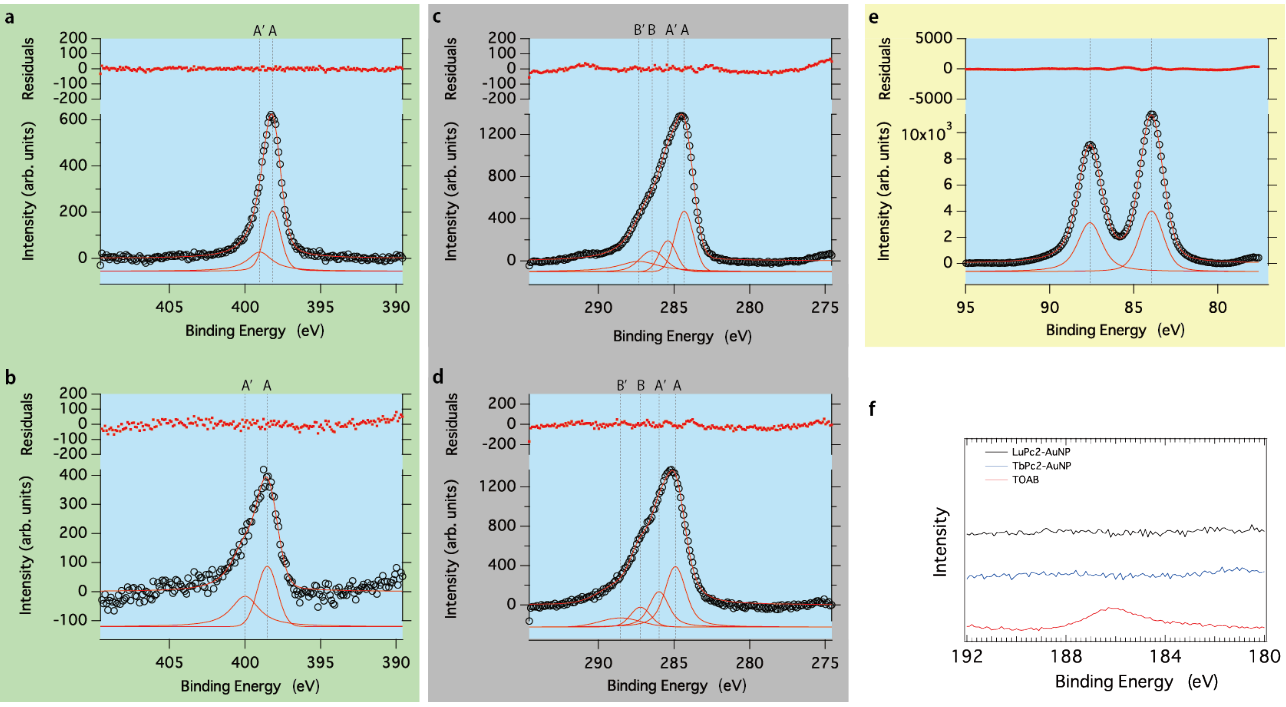

Figure S3 | Photoelectron spectra. (**a**) N 1s of $LuPc_2$, (**b**) N 1s of $LuPc_2$–AuNP, (**c**) C 1s of $LuPc_2$, (**d**) C 1s of $LuPc_2$–AuNP, (**e**) Au 4f of $LuPc_2$, (**f**) Br 3p of $LuPc_2$–AuNP, $TbPc_2$–AuNP and TOAB.

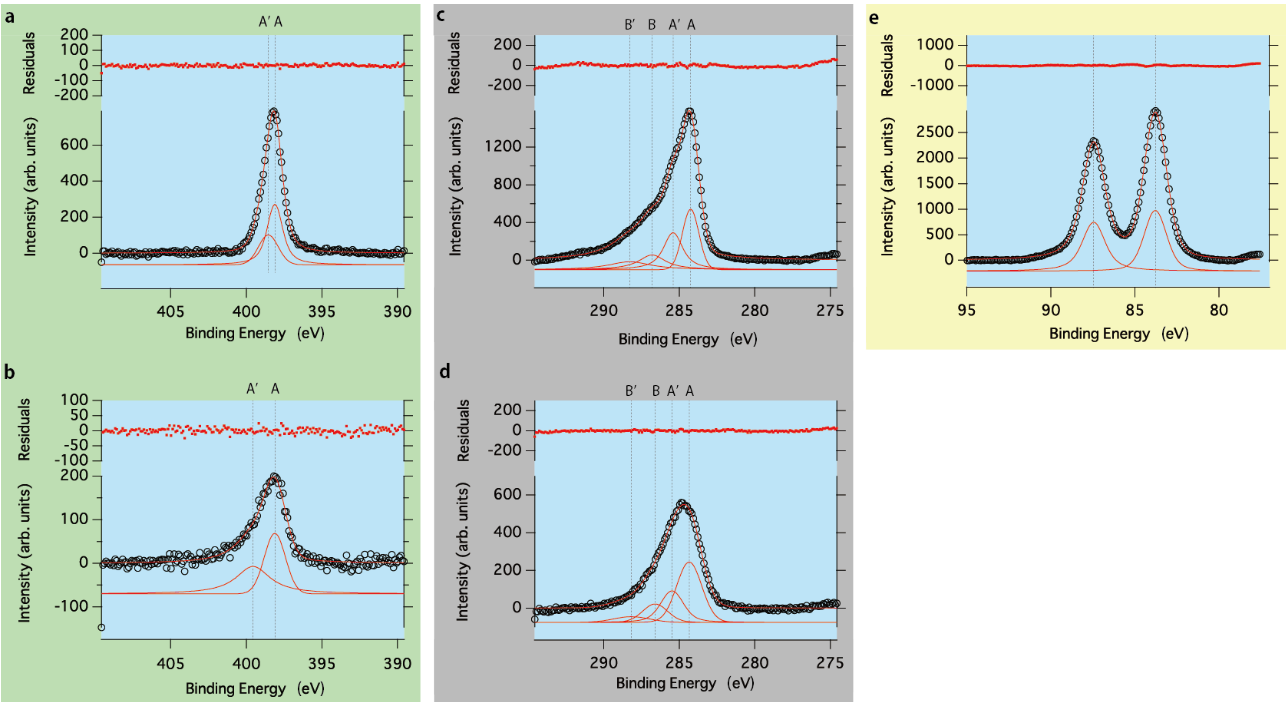

Figure S4 | Photoelectron spectra. (**a**) N 1s of $TbPc_2$, (**b**) N 1s of $TbPc_2$–AuNP, (**c**) C 1s of $TbPc_2$, (**d**) C 1s of $TbPc_2$–AuNP, (**e**) Au 4f of $TbPc_2$.

Table S1 | XPS parameters of the N 1s photopeak in various compounds.

Compound	BE_{main} / eV	$BE_{satellite}$ / eV	Area ratio of main : satellite peak
$LuPc_2$	398.2	399.0	1 : 0.6
$LuPc_2$–AuNP	398.5	400.0	1 : 1.0
$TbPc_2$	398.1	398.5	1 : 0.5
$TbPc_2$–AuNP	398.1	399.6	1 : 1.1

Table S2 | XPS parameters of the C 1s photopeak in various compounds.

Compound	BE_{main} / eV	$BE_{satellite}$ / eV	Area ratio of main : satellite peak
$LuPc_2$	A: 284.3<br>B: 285.4	A′: 286.4<br>B′: 287.3	1 : 0.3
$LuPc_2$–AuNP	A: 284.9<br>B: 285.9	A′: 287.2<br>B′: 288.5	1 : 0.6
$TbPc_2$	A: 284.3<br>B: 285.4	A′: 286.8<br>B′: 288.3	1 : 0.3
$TbPc_2$–AuNP	A: 284.3<br>B: 285.5	A′: 286.6<br>B′: 289.2	1 : 0.6

Table S3 | XPS parameters of the Au $4f_{7/2}$ and Au $4f_{5/2}$ photopeaks in various compounds.

Compound	BE / eV	FWHM / eV
$LuPc_2$–AuNP	83.9<br>87.6	1.64<br>1.69
$TbPc_2$–AuNP	83.8<br>87.5	1.57<br>1.60
bulk Au[8]	84.0<br>87.6	1.02<br>–
Au film on Ti	84.0<br>87.6	1.10<br>–

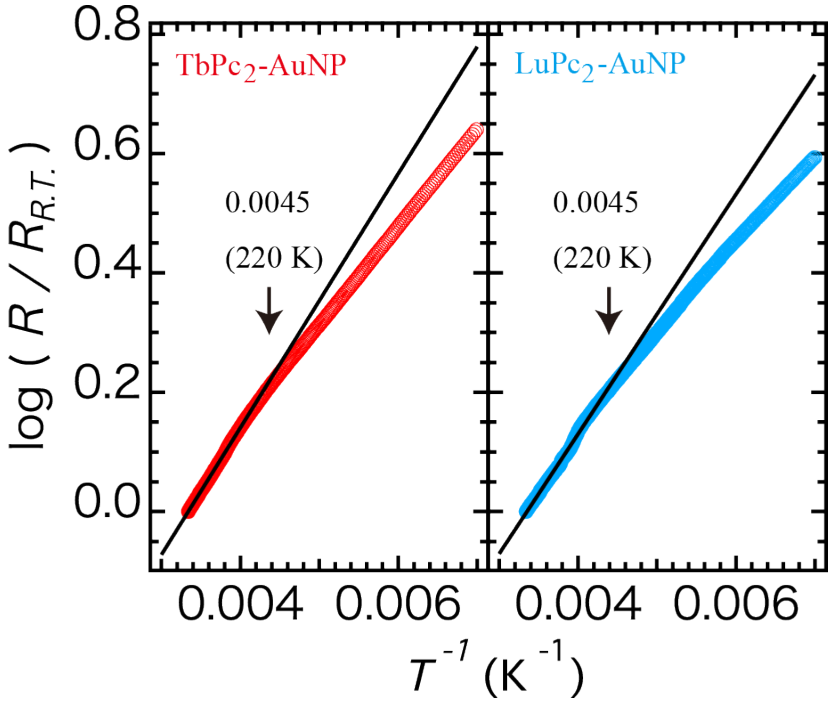

Figure S5| Enlarged view of normalized resistance of $TbPc_2$–AuNP (red) and $LuPc_2$–AuNP(blue) assembly structures plotted vs. T^{-1} shown in Figure 4. The solid lines represent linear fits using Arrhenius models.

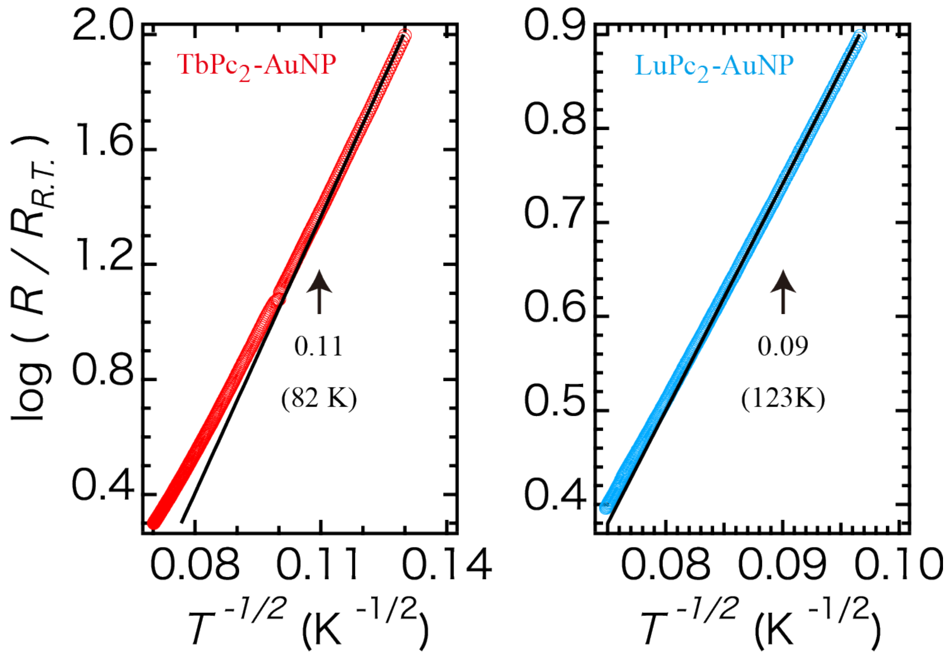

Figure S6 | Enlarged view of normalized resistance of $TbPc_2$–AuNP (red) and $LuPc_2$–AuNP (blue) assembly structures plotted vs. $T^{-1/2}$ shown in Figure 4. The solid lines represent linear fits using ES-VRH models.

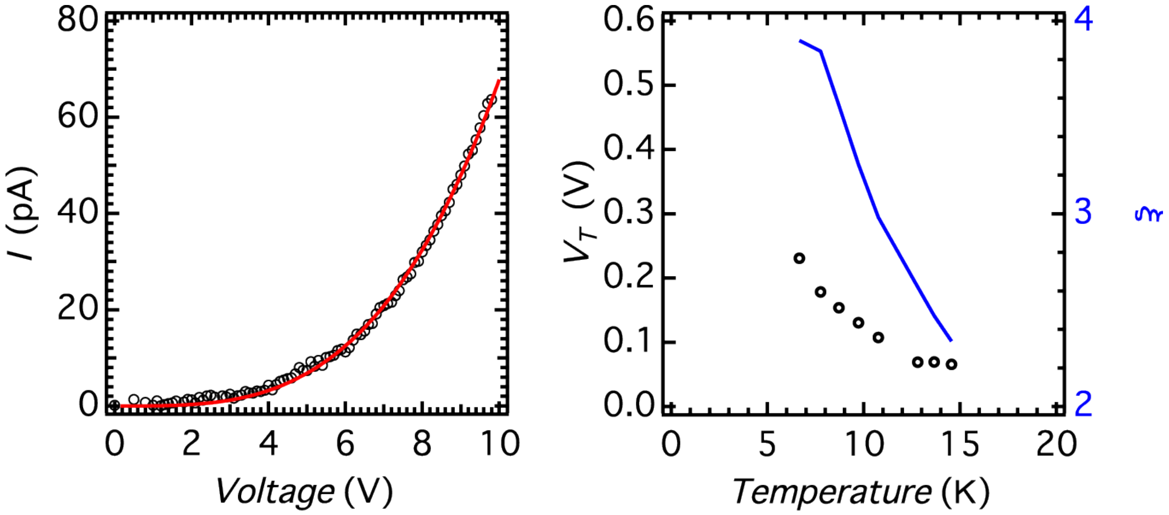

Figure S7 | (a) *I–V* characteristics of a $TbPc_2$–AuNP assembly at 10 K. The red line represents the MW model given by equation (3). The parameters V_T and ξ were 0.13 V and 3.2, respectively. **(b)** Temperature dependence of the parameters V_T and ξ.

---

[1] J. Bufler, M. Abraham, M. Bouvet, J. Simon and W. Gopel, *J Chem Phys* **95**, 8459 (1991).

[2] K. Katoh, Y. Yoshida, M. Yamashita, H. Miyasaka, B. K. Breedlove, T. Kajiwara, S. Takaishi, N. Ishikawa, H. Isshiki, Y. F. Zhang, T. Komeda, M. Yamagishi and J. Takeya, *J Am Chem Soc* **131**, 9967 (2009).

[3] A. J. Signorelli and R. G. Hayes, *J Chem Phys* **64**, 4517 (1976).

[4] Y. Niwa, Kobayash.H and T. Tsuchiya, *J Chem Phys* **60**, 799 (1974).

[5] G. V. Ouedraogo, D. Benlian and L. Porte, *J Chem Phys* **73**, 642 (1980).

[6] X-ray Photoelectron Spectroscopy, edited by Nihon hyomen kagaku kai, japan, 1998 (written in japananese)
